# Changes in Ruminal Fermentation and Growth Performance in Calves After Increasing Ruminal Undegradable Protein at Two Different Time Points Pre-Weaning

**DOI:** 10.3390/ani15060804

**Published:** 2025-03-12

**Authors:** Hamidreza Mirzaei-Alamouti, Sahar Salehi, Mehdi Khani, Mina Vazirigohar, Jörg R. Aschenbach

**Affiliations:** 1 Department of Animal Science, Faculty of Agriculture, University of Zanjan, Zanjan 45371-38111, Iran; 2 Institute of Veterinary Physiology, Freie Universität Berlin, Königsweg 56, 14163 Berlin, Germany; 3 Faculty of Fisheries and Protection of Waters, South Bohemian Research Center of Aquaculture and Biodiversity of Hydrocenoses (CENAKVA), University of South Bohemia in Ceske Budejovice, Zatisi 728/11, 38925 Vodnany, Czech Republic

**Keywords:** rumen-undegradable protein, soybean meal, weaning, calf

## Abstract

The cost-effective rearing of heifers depends on the provision of adequate nutrients at an appropriate age to meet nutrient requirements for growth and production. The objective of the present study was to investigate how the time of switching to a rumen-undegradable protein (RUP) source affects the ruminal fermentation and growth performance of Holstein dairy calves. A total of 36 Holstein female calves were randomly allocated to one of three dietary treatments on day 28 or 42 of life. In the first 28 days of life, they were on the same soybean meal (SBM)-based starter diet containing 22.2% crude protein with 37% RUP. The RUP content of the experimental diet was increased to 48% by a total replacement of the original SBM with xylose-treated soybean meal (XSBM). The following treatments were used: (1) starter diet containing SBM from d 28 (SBM28); (2) starter diet containing XSBM from d 28 (XSBM28); and (3) starter diet containing XSBM from d 42 (XSBM42). All the calves were fed a constant amount of milk (4 L/d), weaned on day 60, and then remained in the experiment until day 70 of life. The results showed that neither feed intake nor BW were influenced by the treatments in the pre-weaning period (days 28 to 60). The calves fed the XSBM28 and XSBM42 diets had greater daily gain and gain-to-feed ratio compared to those on the SBM28 diet post-weaning from day 61 to 70 (*p* < 0.05). The inclusion of XSBM in the starter diets from days 28 and 42 increased the calves’ height pre- and post-weaning (*p* < 0.05). The ruminal pH and concentrations of butyrate and iso-butyrate were higher at weaning when the calves were fed the XSBM diets. The calves fed the XSBM diets had less ammonia-N and total short-chain fatty acids at day 70 of life (*p* < 0.05) and a lower neutral detergent fiber digestibility in the post-weaning period (*p* < 0.05). The fecal scores and blood metabolites were not different among the treatments. The results showed that increasing the RUP content from 37% to 48% in the starter diets by a total replacement of SBM with XSBM during the pre-weaning period may improve the growth performance around weaning. A later replacement at 42 days of life appears equally effective compared to a replacement at 28 days of life.

## Simple Summary

The accelerated growth of calves pre-weaning has positive effects on milk production in the first lactation. To achieve their target growth, calves rely primarily on milk to meet their energy and protein requirements. Around the fourth week, the ruminal fermentation of organic matter and the absorption of fermentation products begin to develop. The solid fermentable feed then provides additional amino acids from its protein sources, primarily soybean meal. To minimize the degradation of soybean meal protein in the rumen and to enhance the direct delivery of amino acids to the intestines, various feed processing techniques have been developed. In the present study, xylose-treated soybean meal was used as a rumen-undegradable protein source in the starter diet of milk-fed calves. It replaced the conventional soybean meal from 28 or 42 days of age. Both approaches resulted in improved daily weight gain and feed efficiency after weaning. The calves fed the xylose-treated soybean meal also exhibited greater body height post-weaning, along with reduced levels of ruminal ammonia nitrogen and short-chain fatty acids. Thus, the present study shows that an increased concentration of rumen-undegradable protein in the pre-weaning starter diet boosts calf performance; however, it seems suitable to introduce the replacement at 42 days of age.

## 1. Introduction

The gastrointestinal tract of newborn calves is not fully developed [1], and they are not able to consume as much solid feed as needed to meet their energy and protein requirements for maintenance and growth. Therefore, they entirely rely on milk or milk replacements with highly digestible nutrients in the first weeks of life [2]. As growing calves gradually consume more starter feed, ruminal fermentation is established, leading to the physical and metabolic development of the rumen [3,4]. Milk feeding strategy, solid feed consumption, and age collectively influence the digestibility of nutrients throughout the digestive tract. Calves receiving greater amounts of liquid feed tend to have lower ruminal digestion due to the way their gastrointestinal tract develops and processes nutrients. Such calves may struggle to digest dry matter efficiently and to meet their nutritional needs around weaning. In contrast, calves fed a restricted milk diet consume more solid feed, which promotes rumen development and enhances fiber digestibility [5].

Optimized ruminal fermentation, sufficient ruminal volume, as well as ruminal absorptive capacity, usually establish after 4 to 6 weeks of age [6,7]. Before weaning, the neonate requires much energy and protein [8]. Supplemented protein can provide amino acids (AAs) which are essential for the maintenance and growth of dairy calves [9]. Among all protein sources, soybean meal (SBM) is used most commonly [10] because of the high proportion of essential AAs. However, soybean meal is easily degraded in the rumen which, together with its content of anti-nutritive factors and allergenic proteins, may limit its use in pre-weaning calf diets [10]. When the ruminal degradation of true protein is high, microbes cannot utilize all the ammonia released. The excess ammonia then enters the bloodstream, with a substantial portion not being recycled to the rumen; instead, it is excreted as urea in the urine. This process is not only energy-consuming but has additional environmental consequences. Therefore, various chemical and thermo-mechanical processing methods have been developed to increase the nutritional value and post-ruminal absorption of amino acids from SBM.

The heat treatment of soybean meal (SBM) is a widely used method to lower ruminal protein degradability and enhance the flow of dietary amino acids (AAs) to the small intestine [11]. Achieving this requires precise control of the heating process. Chemical treatment with lignosulfonate [12,13], tannins [14], bentonite [15], formaldehyde [16,17], and other chemical agents [16] are other ways to increase the flow of dietary AAs to the small intestine. However, some of these chemical treatments do not enhance the total flow of essential AAs due to a reduction in the microbial protein flow [16]. An effective alternative is the combined use of heat and xylose to better control the Maillard reaction between sugar aldehyde groups and amino groups [18]. For xylose-treated SBM, it has been shown that this method can decrease the ruminal degradability of soybean meal proteins by about 86% without impeding intestinal protein digestion [19,20].

The provision of rumen-undegradable protein (RUP) in adult ruminants improved the performance and efficiency of nitrogen (N) utilization [21], followed by the enhanced flow of N and AA to the small intestine [22]. At first glance, the differentiation into rumen degradable protein (RDP) and RUP may appear less relevant for the starter feed of pre-weaning calves due to an underdeveloped rumen. However, fermentation gradually becomes significant during ruminal development, as evidenced by the already high short-chain fatty acid (SCFA) concentrations (>100 mM) in the rumens of calves before weaning [23,24]. Nonetheless, the results regarding the use of RUP sources in the starter diets of calves are controversial. Some researchers have found the positive effects of enhanced RUP on performance and feed efficiency, as well as a reduced incidence and severity of diarrhea [22,25,26]. Furthermore, reducing dietary crude protein to 16% of the dry matter while increasing RUP to 6.5% of the dry matter did not negatively affect calf performance [27], suggesting that increasing RUP can enhance amino acid delivery to the small intestine. Yousefinejad et al. [24] demonstrated that increasing RUP concentration had no significant impact on calf growth before weaning but stimulated growth after weaning. Maiga et al. [28] found that increasing the RUP level could enhance dry matter intake, as well as growth hormone and insulin-like growth factor-1 concentrations in the blood plasma. On the other hand, others found no improvement in calf growth in response to feeding with various RUP levels [25,26,29]. The latter may be attributed to the incomplete development of the rumen where exchanging RUP for RDP may not alter the amount and type of AAs flowing to the intestine, as inferred from a study by Tahmasbi et al. [30]. Considering that the latter study was performed in very young calves (from birth up to 6 weeks of age), these findings could indicate that the effects of RUP in starter feed may be age dependent. The ruminal fermentation and capacity for absorption develop until approximately 4 to 6 weeks after birth [6,7], justifying the hypothesis that the age at which calves consume RUP might explain the variable responses in the previous studies. To date, no specific recommendations have been made regarding the RUP content in the starter diets for milk-fed calves [31]. Moreover, most studies have focused on total CP concentration without specific reference to the RDP and RUP fractions of the starter diets used.

Based on the results of previous studies, we hypothesized that increasing RUP in the starter diet could alter ruminal fermentation and improve growth performance but that the timing of RUP provision may be critical. Thus, the present study was designed to evaluate the effects of replacement of RDP (SBM) from the same SBM source in the starter diet with RUP (XSBM) from either 28 or 42 days of life on Holstein dairy calves. The latter time points were chosen because they represent the time where milk feeding peaks in most feeding programs, implying that solid feed intake gradually becomes relevant for both nutrient supply and ruminal development. Target variables were ruminal fermentation and health and growth performance during the pre- and post-weaning periods.

## 2. Materials and Methods

All animal care and managerial procedures were aligned to protocols approved by the Iranian Council of Animal Care. The Animal Care and Welfare Committee at the University of Zanjan, Iran, approved the experimental and management protocols of this study “(ID: 1353)”. The experiment was carried out from March to June 2017 at a commercial dairy farm in Ghazvin, Iran.

### 2.1. Calves, Treatments, and Management

A total of 36 Holstein female dairy calves in good health condition were included in the experiment at either 28 or 42 days of age with starting body weights (BW) of 42 ± 2.1 and 54 ± 2.4 kg, respectively. The calves had been separated from their dams immediately after birth and housed in individual pens bedded with straw. The bedding was renewed every 24 to 48 h as required. Calves were fed 4 L/day of whole milk (containing 3.6 ± 0.34% milk fat, 3.20 ± 0.11% protein, 4.67 ± 0.03% lactose, and 11.61 ± 0.11% total solids) in two equal portions at 8:00 and 17:00 h from d 3 to 56, followed by a morning feeding of 2 L/day from 57 to 60 day of age. All the calves were weaned at day 61 of age and remained in the study until day 70 of age. They had free access to water throughout the study period. The calves were fed a starter diet (RUP = 37%) based on corn and SBM until day 28 of life. Thereafter, 12 calves were switched to a starter diet in which SBM (RUP = 30%) was completely replaced with XSBM (RUP = 65%), with the aim of increasing dietary RUP to 48%. On day 42, another 12 calves were switched from the SBM to the XSBM diet. This resulted in the random assignment to the following three treatment groups: (1) starter diet containing SBM that was offered from day 28 onwards (SBM28), (2) starter diet containing XSBM that was offered from day 28 onwards (XSBM 28), and (3) starter diet containing XSBM that was offered from day 42 onwards (XSBM42). The diets were formulated as recommended by NRC 2001 [11] to meet the calves’ energy and protein requirements. The diets had the same ingredients and similar nutrient compositions, with the main exception that the total replacement of SBM with XSBM resulted in different RUP and RDP contents (Table 1). There was also a small discrepancy in the dietary phosphorous concentration among the groups that was unintended.

### 2.2. Feed Intake, Performance, and Skeletal Growth Parameters

Feed was offered to each calf daily, and the orts were collected to calculate the daily feed intake. All the feed buckets were checked in the afternoon throughout the study and changed if required. The feed samples were collected and stored at −20 °C until further chemical analysis. Individual BW was recorded at 28, 42, 60, and 70 days of age using an electronic scale. The skeletal growth parameters, including wither height (distance from the base of the front feet to the withers), body length (distance between the points of the shoulder and rump), hip height (distance from the base of the rear feet to hook bones), and hip width (distance between the points of hook bones), were also measured at the time of weighting.

### 2.3. Ruminal Sampling and Chemical Analysis

The ruminal fluid was collected 3–4 h after the morning feeding using a stomach tube fitted to a vacuum pump on days 60 and 70. The ruminal pH was measured immediately using a pH meter (HI 8314 membrane pH meter; Hanna Instruments, Villafranca, Italy). The ruminal fluid samples were squeezed through four layers of cheesecloth, then 8 mL of the ruminal fluid was acidified with 2 mL of 25% meta-phosphoric acid and stored at −20 °C until the analyses of short-chain fatty acids (SCFAs) and ammonia-N. To analyze the concentrations of SCFA, the acidified ruminal fluid samples were thawed, shaken, and allowed to settle at room temperature for 15 min. A 5 mL aliquot of the ruminal fluid supernatant and 1 mL of the meta-phosphoric acid internal standard (2-ethyl butyric acid; Sigma-Aldrich, St. Louis, MO, USA) solution were mixed and transferred into a 15 mL glass test tube. The tube was centrifuged at 12,000× *g* and 4 °C for 15 min. The prepared samples were transferred into an Eppendorf tube and a 1 μL aliquot of the upper layer was injected into a gas chromatograph (Varian 3400; Varian Inc., Walnut Creek, CA, USA) equipped with an injector at 170 °C, a flame-ionization detector at 175 °C, and a packed column (6’ × 2 mm ID glass containing 1-1965 10% SP-1200/1% H_3_PO_4_ on 80/100 Chromosorb W). The temperature of the gas chromatograph oven was isothermal and maintained at 140 °C. Gas flow rates were nitrogen 40 mL/min and compressed air 300 mL/min [32]. To determine the concentration of ammonia-N in the ruminal fluid samples, the procedure of Broderick and Kang [33] was used with the minor modification in which manganese sulfate was used as a catalyst.

### 2.4. Blood Sampling and Biochemical Measurements

Blood samples were collected from the jugular vein into 10 mL tubes on days 28, 42, 60, and 70 of life 3 h after the morning feeding. They were immediately centrifuged at 3000× *g* at 4 °C for 15 min to separate the plasma, which was then stored at −20 °C for subsequent analysis. The blood metabolite concentrations including glucose, albumin, calcium, total protein, plasma nitrogen urea, and globulin were determined using commercially available kits in accordance with the manufacturer’s instructions.

### 2.5. Fecal Consistency Scoring and Sampling

The physical shape and fecal consistency were scored once daily before the morning feeding according to a 5-point scale as follows: 1 = normal; 2 = soft to loose; 3 = loose to watery; 4 = watery, mucous, slightly bloody; 5 = watery, mucous, and bloody. The fecal samples were collected from the rectum of each calf on 28, 42, 60, and 70 days of age in the morning and afternoon before offering the new feed. The samples were placed into plastic containers and stored at −20 °C until analysis.

### 2.6. Feed and Digestibility Analysis

Representative samples of the starter diet, orts, and feces were dried at 55 °C for 72 h to determine the dry matter content and then ground through a 1 mm screen for the subsequent analysis of CP, ether extract, and ash according to the standard procedures described by AOAC [34]. Neutral detergent fiber (NDF) was determined using the method described by Van Soest et al. [35]. Apparent total tract digestibility of nutrients was measured using acid-insoluble ash (AIA) as an internal marker [36]. The RUP and RDP values of the soybean meal and xylose-treated soybean meal were measured using an in situ technique. Briefly, 5 g of soybean meal was placed in 50 μm pore-size nylon bags and incubated for 48 h in the rumen of three fistulated non-lactating Holstein cows (body weight = 627 kg). The cows were fed twice a day on an 80 % forage and 20 % concentrate diet. The protein disappearance from the bags was measured over 48 h, and the resulting data were fitted to a nonlinear model to determine the quantity of RUP and RDP.

### 2.7. Statistical Analysis

Data were tested for normality by using PROC UNIVARIATE and were analyzed using the MIXED procedure of SAS (Version 9.4; SAS Institute Inc., Cary, NC, USA). The statistical model was Y_ik_ = µ + D_i_ + C_k_(D_i_) + e_ik_, where Y_ik_ is the dependent variable, µ is the overall mean, D_i_ is the fixed effect of dietary treatment i, C_k_(D_i_) is the random effect of calf nested in the dietary treatment, and e_ik_ is the residual error.

The variables measured in the experiment were divided into three time periods, including 28–42, 43–60, and 61–70 days of age. For each of the variables analyzed, the calves nested within each treatment group were subjected to the following three covariance structures: compound symmetric, autoregressive order one, and unstructured covariance. A variance–covariance structure was chosen based on the best Akaike information criterion. Mean comparisons were performed by the least square mean (LSM) method, and the differences were compared using Tukey test. To avoid any bias in variance parameters, the effects of the initial values were added as covariates to the model. The covariates were removed from the model one at a time if its significance probability was >0.1, starting with the least significant and in a backward stepwise manner. The significance and tendency for differences between the treatments were determined at *p* < 0.05 and 0.05 ≤ *p* < 0.10, respectively. Data are expressed as LSM ± standard error of mean (SEM), unless otherwise stated.

## 3. Results

### 3.1. Starter Intake, Daily Gain, and Gain-to-Feed Ratio

Starter intake, average daily gain, BW, as well as gain-to-feed ratio, were not different at 42 and 60 days of life (Table 2). However, in the post-weaning period, the average daily gain and the gain-to-feed ratio were significantly higher for the calves fed the XSBM diets compared to those consuming the SBM diet (*p* ˂ 0.05).

### 3.2. Skeletal Growth

Based on the length and height measurements, the calves fed the XSBM diets were taller and longer than the calves fed the SBM diet during the pre- and post-weaning periods (Table 3). On day 42 of age, the hip height for the calves fed XSBM28 was higher than the calves that consumed the SBM diet (*p* ˂ 0.01). On day 60 of life, the body length and the hip and wither height for the calves fed XSBM28 and XSBM42 were greater than for the calves fed the SBM diet (*p* ˂ 0.01). On day 70 of life, the wither height was greater for both XSBM diets than the SBM diet (*p* ˂ 0.01).

### 3.3. Ruminal Fermentation Profile

On day 60, the calves fed the XSBM diet from 42 days of age had a lower ruminal pH (*p* < 0.01) than the calves fed SBM (Table 4). Furthermore, the calves on both XSBM diets tended to have lower total SCFA concentrations (*p* = 0.09) with lower iso-valerate but higher butyrate and iso-butyrate concentrations than the calves on the SBM diet (*p* ˂ 0.05).

On day 70 of age, the ruminal ammonia-N (*p* < 0.01) and total SCFA (*p* < 0.05) concentrations were higher in the calves fed the SBM diet than the calves on XSBM (*p* ˂ 0.05) (Table 4).

### 3.4. Fecal Consistency and Total Tract Nutrient Digestibility

The fecal consistency was not influenced among treatments from day 42 through day 60 of age (*p* > 0.1, Table 5). Except for increased NDF digestibility in the SBM group at 70 days of age (*p* < 0.05), none of the nutrient digestibility values were influenced by the diets through the entire experiment (*p* > 0.1, Table 5).

### 3.5. Plasma Metabolites

The dietary treatments did not affect the blood metabolite concentrations during the experimental period (*p* > 0.1; Table 6). On day 60 of life, the total plasma protein content tended to be higher in the calves fed the XSBM diets compared to those on the SBM diet (*p* = 0.08).

## 4. Discussions

The aim of the present study was to examine how an increased RUP concentration in the starter diet, either from 28 or 42 days of life, would impact the ruminal fermentation and performance of Holstein dairy calves. The hypothesis that the timing of RUP provision may be critical was derived from the knowledge regarding the development of ruminal fermentation. The establishment of a preliminary ruminal microbial ecosystem begins shortly after birth [37], long before the rumen becomes fully developed [38]. However, it is reasonable to assume that ruminal fermentations might not become a priority until 4 weeks of age due to an active reticular groove and low starter intake [6]. Thereafter, the functionality of the rumen typically develops between approximately 4 and 6 weeks of age [6,7]. It can thus be assumed that supplementing RUP sources in the diet from either 4 weeks or 6 weeks of age onwards may has the potential to increase nitrogen and AA flow to the small intestine. Intestinal AA provision, in turn, has been shown to enhance growth performance [39]. One effective way to reduce ruminal protein degradation and increase post-ruminal availability of AAs is the processing of soybean meal with xylose [40]. This processing approach was chosen for the present study instead of sole heat processing. The challenge with heat processing is that the temperature and duration of heating must be carefully chosen to control the Maillard reaction and to prevent a reduction in the intestinal digestibility of RUP [10]. By contrast, xylose treatment during the heat protocol enables an optimized control of the Maillard reaction and thus enables optimal post-ruminal digestibility of RUP [20].

The starter intake remained unaffected by both XSBM treatments throughout the study. This is contrary to the findings by Kazemi-Bonchenari et al. [26], who observed decreased dry matter intake when SBM was replaced with XSBM. Another study by ZeidAli-Nejad et al. [41], using extruded full-fat soybean as an RUP source, also observed a small decrease in starter intake post-weaning. In both the previous studies, however, no significant differences in BW were observed when the RUP levels were increased. Body weight was also not affected significantly during the entire period of our present study. Nonetheless, the average daily gain and the gain-to-feed ratio were higher for the calves fed the XSBM diets in the final period of measurements post-weaning. The latter findings are consistent with the data reported by Maiga et al. [28], who found a higher average daily gain when soybean meal was replaced by extruded SBM. Another study demonstrated that average daily gain and feed efficiency were higher with a high RUP diet [30]. Together, all these studies point to an optimized performance after RUP provision due to either decreased feed intake with similar performance or constant feed intake with increased body weight gain. This may be attributed to the greater post-ruminal availability of AAs for absorption and a higher concentration of plasma insulin [24].

It needs to be acknowledged, however, that not all the studies reported production benefits when partially replacing RDP with RUP. For example, Kazemi-Bonchenari et al. [26] found no difference in daily gain and feed efficiency when using XSBM instead of regular SBM. Therefore, the calves’ responses to RUP in the starter diets likely depend on additional factors, including the milk-feeding program, weaning method, feed protein source and processing technique, and calf age, all of which are known to affect the nitrogen metabolism in the rumen. The present study adds the conclusion that the timing of RUP provision is likely not among those additional factors, as long as the introduction of RUP occurs no later than 42 days of life.

The higher daily gain in the present study was paralleled by improvements in the majority of skeletal growth parameters due to the XSBM diets. The observed enhancement of skeletal growth in the calves receiving a high-RUP diet is consistent with the findings of Moallem et al. [42], who observed higher responsiveness of skeletal growth variables to RUP levels before 90 d of age. Similarly, Kazemi-Bonchenari et al. [43] reported improvements in skeletal growth variables with increased RUP. Again, this phenomenon is likely attributable to the greater availability of AAs in the small intestine resulting from the elevated RUP content of XSBM diets and possibly also positive effects on plasma insulin concentration [24].

The treatments applied during the pre-weaning period had an effect on ruminal pH at day 60. Reductions in pH appear to be closely linked to feeding times, the ruminal digestibility and amount of organic matter, and the absorption rate of short chain fatty acids by the ruminal epithelium [44]. In our study, we observed that calves fed the SBM diet had higher ruminal pH compared to those receiving XSBM until day 60. A higher pH in the rumen may create a more favorable ruminal environment for cellulolytic bacteria to thrive, as indicated by the higher NDF digestibility in the calves fed the SBM diet and a trend for higher concentrations of total SCFA. However, no significant differences were observed in the ruminal concentrations of acetate and propionate, as well as in the acetate-to-propionate ratio before and after weaning, indicating that the metabolic advantage from this type of fermentation may be limited. Additionally, our findings indicate a significant increase in ruminal branched-chain fatty acids (BCFA), such as iso-valerate, with the SBM diet, which is known to correlate with cellulolytic bacteria activity [45]. Similarly, other studies have shown that supplementing dietary BCFAs leads to increased nutrient digestibility, ruminal SCFA concentration, and cellulolytic bacteria [46,47]. However, our results differ from those of Rastgoo et al. [48], ZeidAli-Nejad et al. [41], and Yousefinejad et al. [24], who found no significant differences in pH and total SCFA concentration. These discrepancies may be attributable to variations in the source and level of RUP, the AA profiles of feedstuffs, and the interactions with other nutrients [49,50]. Furthermore, the ruminal concentration of NH_3_-N was higher for the SBM treatment on d 70, indicating that increased dietary RUP content reduced ruminal protein degradation and, hence, ruminal ammonia-N concentration [51].

As mentioned, the apparent total tract digestibility of NDF showed lower values for XSBM compared to the SBM treatment on the last day of experiment. This can be attributed to the lower degradability of the XSBM protein, leading to decreased microbial activity, thereby lowering the digestibility of feed nutrients [52]. Reducing RDP in the XSBM diet, and consequently decreasing ruminal amino acids, peptides, and BCFAs, may have led to reduced fiber digestion. The effects of these substrates on fiber digestion have been recently reviewed [47]. The fecal consistency scores did not show any variation between the treatments over the course of the investigation, aligning with findings of Kazemi-Bonchenari et al. [26].

The present study also monitored the selected plasma indicators of energy and protein homeostasis in the blood of the experimental animals. The regulation of blood glucose levels in ruminants predominantly hinges on gluconeogenesis, a metabolic pathway where propionate acts as the primary precursor for glucose synthesis [53]. Moreover, dry matter intake assumes a crucial role, as increased feed consumption is associated with elevated blood glucose concentrations [54]. In the present study, the plasma glucose concentrations remained unchanged, aligning with the findings that dietary interventions had no impact on feed intake or ruminal propionate concentration. Plasma total protein tended to exhibit a small increase in the XSBM diets on day 60 of life, which might be related to the type of protein supply [55], pointing to superior protein accretion efficiency with the XSBM diets relative to the SBM diet. Higher numerical plasma total protein concentration may suggest enhanced liver function and greater amino acid availability for the immune system and calf growth during the transition to solid feed, potentially improving future production performance. However, the other plasma indicators of protein metabolism, including plasma urea nitrogen concentrations, were not altered.

When finally considering the positive effects of the XSBM vs. SBM diets on animal performance in the present study, we need to acknowledge that those two diets did not only differ in RUP but also in phosphorous content. The latter was not intended but became obvious during the feed analysis. Thus, the question arises whether the lower content of phosphorous could have contributed to the positive effects of the XSBM diet. The latter can be clearly refused. In a recent study, we showed that an increase in dietary phosphorous concentration from 0.4% to 0.8% greatly promoted the daily gain of Holstein calves on a forage-containing starter diet around weaning [56]. The composition of the alfalfa forage-containing diet in that previous study was quite similar to the diet composition in the present study. As such, the positive effects of the XSBM diet in the present study cannot be attributed to an obviously lower dietary phosphorous concentration but occurred despite the lower phosphorous concentration. We suggest that the likely interaction between dietary RUP and phosphorous concentrations on calf performance should be investigated in detail in future studies. Furthermore, long-term studies are required to evaluate the impact of starter diet RUP on calf health and its influence on future milk production.

## 5. Conclusions

Providing XSBM to pre-weaning calves seems to offer a promising approach for enhancing dairy calf performance by providing increased post-ruminal AAs and energy, facilitated by efficient nitrogen utilization. A complete substitution of SBM with XSBM appears to provide potential benefits without adversely impacting ruminal fermentation or growth performance. The beneficial effects were observed to be independent of the period of RUP provision, indicating that the implementation of RUP from 42 days onwards was similarly effective to an earlier implementation from 28 days of life. These time points indicate the period in which the rumen develops its functionality.

## Figures and Tables

**Table 1:** Ingredients and chemical composition of experimental diets.

Items	Diet ^1^	
	SBM	XSBM
Ingredients, % of DM		
Alfalfa hay	9.25	9.25
Ground barely grain	10.19	10.19
Ground corn grain	39.82	39.82
Xylose-treated soybean meal	-	31.48
Soybean meal	31.48	-
Roasted soybean meal	1.85	1.85
Corn germ	2.41	2.41
Calcium carbonate	0.46	0.46
Sodium bicarbonate	0.93	0.93
Salt	0.46	0.46
Vitamin and mineral mix ^2^	2.78	2.78
Chemical composition, % of DM		
Dry matter	94.6	94.4
Crude protein	22.2	22.1
Rumen-degradable protein	63.0	51.9
Rumen-undegradable protein	37.1	48.1
Neutral detergent fiber	19.8	19.0
Non-fiber carbohydrate ^3^	50.2	50.5
Ether extract	2.2	2.2
Calcium	0.85	0.87
Phosphorous	0.45	0.37
ME (Mcal/kg) ^3^	2.75	2.75

^1^ SBM, soybean meal (RUP = 30% of CP); XSBM, xylose-treated soybean meal (RUP = 65% of CP); RUP, rumen-undegradable protein. ^2^ Containing per kilogram of supplement: 1,500,000 IU vitamin A, 350,000 IU vitamin D3, 8000 IU vitamin E, 400 mg antioxidant, 150 g Ca, 30 g Mg, 10 g Mn, 20 g Zn, 5 g Cu, 70 mg Co, 100 mg Se, 120 mg I. ^3^ Calculated according to NRC 2001 [11]; Non-fiber carbohydrate = 100 − (% NDF + % CP + % EE + % Ash), ME = (((1.01 × DE) − 0.45) + 0.0046) × (EE − 3).

**Table 2:** Starter intake, body weight, average daily gain, and gain-to-feed ratio in peri-weaning dairy calves fed xylose-treated soybean meal from 28 or 42 days of life.

Items	Diet ^1^			SEM	*p*-Value
	XSBM42	XSBM28	SBM		
Period 28–42 days	-				
Starter intake (g/d)	-	707	732	60.4	0.77
Body weight at d 28 (kg)	-	44.9	44.2	0.94	0.58
Body weight at d 42 (kg)	-	54.9	52.6	1.46	0.27
Average daily gain (g/d)	-	818	724	58.1	0.26
Gain-to-feed ratio	-	1.2	1.02	0.15	0.3
Period 43–60 days					
Starter intake (g/d)	1122	1104	1109	75.4	0.81
Body weight at d 42 (kg)	54.2	54.9	52.6	1.15	0.11
Body weight at d 60 (kg)	70.3	70.9	67.5	2.31	0.15
Average daily gain (g/d)	891	880	820	68.1	0.27
Gain-to-feed ratio	0.79	0.81	0.73	0.07	0.14
Period 61–70 days					
Starter intake (g/d)	1850	1852	1878	106.9	0.98
Final body weight at d 70 (kg)	81	80.8	77.2	2.42	0.21
Average daily gain (g/d)	1001 ^a^	1004 ^a^	958 ^b^	46.2	0.03
Gain-to-feed ratio	0.57 ^a^	0.54 ^a^	0.51 ^b^	0.041	0.04

^1^ SBM, soybean meal; XSBM28, Xylose-treated soybean meal fed from 28 days of life; XSBM42, Xylose-treated soybean meal fed from 42 days of life. ^a,b^ Means with a different superscript denote the significant difference (*p* < 0.05).

**Table 3:** Effects of whole replacement of xylose-treated soybean meal for soybean meal in starter feed from 28 or 42 days of life on skeletal growth indices of Holstein calves.

Items	Diet ^1^			SEM	*p*-Value
	XSBM42	XSBM28	SBM		
42 days					
Body length (cm)	-	77.1	77.1	0.54	0.98
Hip height (cm)	^-^	65	61.8	0.85	0.01
Wither height (cm)	^-^	83.2	82	0.55	0.13
Hip width (cm)	-	24.9	25.5	0.21	0.18
60 days					
Body length (cm)	79.8 ^a^	79.1 ^a^	78.1 ^b^	1.64	0.003
Hip height (cm)	67.2 ^a^	67.9 ^a^	62.1 ^b^	0.95	0.002
Wither height (cm)	91.3 ^a^	87.7 ^a^	82.2 ^b^	0.91	0.001
Hip width (cm)	26.3	26	26	0.22	0.61
70 days					
Body length (cm)	83	81.8	82.4	1.11	0.71
Hip height (cm)	69.6	69.5	67.3	0.86	0.11
Wither height (cm)	93.0 ^a^	91.8 ^a^	88.5 ^b^	0.61	0.001
Hip width (cm)	26.5	26.1	26.3	0.22	0.42

^1^ SBM, soybean meal; XSBM28, xylose-treated soybean meal fed from 28 days of life; XSBM42, xylose-treated soybean meal fed from 42 days of life. ^a,b^ Means with a different superscript denote the significant difference (*p* < 0.05).

**Table 4:** Effects of whole replacement of xylose-treated soybean meal for soybean meal in starter feed from 28 or 42 days of life on ruminal fermentation characteristics of Holstein calves.

Items	Diet ^1^			SEM	*p*-Value
	XSBM42	XSBM28	SBM		
60 days					
pH	6.35 ^b^	6.58 ^ab^	6.96 ^a^	0.072	0.001
NH_3_-N (mg/dL)	18.5	16.9	17.3	1.08	0.51
Total SCFA (mmol/L)	71.3	71.4	79.6	6.15	0.09
Acetate (mol/100 mol)	43.8	44.5	45	1.34	0.21
Propionate (mol/100 mol)	39.6	36.5	39.6	1.26	0.45
Butyrate (mol/100 mol)	11.1 ^a^	11.4 ^a^	8.7 ^b^	0.85	0.049
iso-Butyrate (mol/100 mol)	0.51 ^a^	0.51 ^a^	0.37 ^b^	0.021	0.021
Valerate (mol/100 mol)	5.23	5.62	5.53	0.631	0.14
iso-Valerate (mol/100 mol)	0.63 ^b^	0.68 ^b^	0.73 ^a^	0.072	0.046
Acetate-to-propionate ratio	1.11	1.23	1.35	0.13	0.14
70 days					
pH	6.32	6.91	6.43	0.08	0.14
NH_3_-N (mg/dL)	13.4 ^b^	15.3 ^ab^	18.4 ^a^	1	0.009
Total SCFA (mmol/L)	68.7 ^b^	68.8 ^b^	82.7 ^a^	0.73	0.022
Acetate (mol/100 mol)	44.6	48.2	50.3	1.8	0.11
Propionate (mol/100 mol)	38.2	35.2	36.8	1.81	0.52
Butyrate (mol/100 mol)	5.95	6.6	6.03	0.512	0.64
iso-Butyrate (mol/100 mol)	0.41	0.53	0.36	0.022	0.22
Valerate (mol/100 mol)	8.05	7.18	5.82	1.421	0.13
iso-Valerate (mol/100 mol)	0.86	0.66	0.71	0.071	0.14
Acetate-to-propionate ratio	1.17	1.38	1.37	0.12	0.15

^1^ SBM, soybean meal; XSBM28, xylose-treated soybean meal fed from 28 days of life; XSBM42, xylose-treated soybean meal fed from 42 days of life. ^a,b^ Means with a different superscript denote the significant difference (*p* < 0.05).

**Table 5:** Effects of whole replacement of xylose-treated soybean meal for soybean meal in starter feed from 28 or 42 days of life on nutrient digestibility and fecal score of Holstein calves.

Items	Diet ^1^			SEM	*p*-Value
	XSBM42	XSBM28	SBM		
42 days					
Dry matter digestibility	-	77.1	77.3	1.47	0.92
Organic matter digestibility	-	78.6	78.9	1.49	0.81
Crude protein digestibility	-	77.8	75.7	1.76	0.41
Neutral detergent fiber digestibility	-	41.2	40.7	3.95	0.91
Fecal score	-	1	1.05	0.01	0.17
60 days					
Dry matter digestibility	74.9	76.3	79	4.09	0.37
Organic matter digestibility	77.4	78	80.1	4.02	0.52
Crude protein digestibility	75.2	78	80	4.26	0.28
Neutral detergent fiber digestibility	43.6	50	55.2	9.92	0.61
Fecal score	1.52	1.04	1.02	0.18	0.12
70 days					
Dry matter digestibility	73.3	77.9	80.7	3.58	0.12
Organic matter digestibility	75.5	78.7	82.2	3.52	0.15
Crude protein digestibility	75.8	80.7	81.6	3.41	0.22
Neutral detergent fiber digestibility	46.0 ^c^	51.1 ^b^	61.8 ^a^	7.95	0.04
Fecal score	1.5	1.03	1.02	0.211	0.21

^1^ SBM, soybean meal; XSBM28, xylose-treated soybean meal fed from 28 days of life; XSBM42, Xylose-treated soybean meal fed from 42 days of life. ^a,b,c^ Means with a different superscript denote the significant difference (*p* < 0.05).

**Table 6:** Effects of whole replacement of xylose-treated soybean meal for soybean meal in starter feed from 28 or 42 days of life on blood parameters of Holstein calves.

Items	Diet ^1^			SEM	*p*-Value
	XSBM42	XSBM28	SBM		
42 days					
Glucose (mg/dL)	-	98.6	97	2.49	0.66
Albumin (g/dL)	-	4.08	4.06	0.034	0.72
Calcium (mg/dL)	-	8.71	8.59	0.051	0.13
Total protein (g/dL)	-	6.05	6.08	0.038	0.68
Plasma urea nitrogen (mg/dL)	-	18.8	18.5	0.14	0.17
Globulin (g/dL)	-	1.97	2.01	0.058	0.65
60 days					
Glucose (mg/dL)	88.3	93.9	94.3	2.02	0.88
Albumin (g/dL)	4.22	4.07	4.09	0.101	0.15
Calcium (mg/dL)	8.54	8.71	8.69	0.043	0.99
Total protein (g/dL)	6.21	6.22	6.12	0.072	0.081
Plasma urea nitrogen (mg/dL)	18.6	19.3	18.8	0.37	0.12
Globulin (g/dL)	1.84	2.14	2.01	0.137	0.32
70 days					
Glucose (mg/dL)	75.8	73.8	76.1	0.83	0.71
Albumin (g/dL)	4.24	4.22	4.21	0.094	0.58
Calcium (mg/dL)	8.77	8.45	8.39	0.158	0.43
Total protein (g/dL)	6.18	6.26	6.21	0.062	0.48
Plasma urea nitrogen (mg/dL)	19.8	19.6	19.5	0.55	0.91
Globulin (g/dL)	1.78	2.04	2.01	0.101	0.52

^1^ SBM, soybean meal; XSBM28, xylose-treated soybean meal fed from 28 days of life; XSBM42, xylose-treated soybean meal fed from 42 days of life. Means with a different superscript denote the significant difference (*p* < 0.05).

## Data Availability

The data presented in this study are available on request from the corresponding author.
